# Aryl Hydrocarbon Receptor Activation Produces Heat Shock Protein 90 and 70 Overexpression, Prostaglandin E2/Wnt/β-Catenin Signaling Disruption, and Cell Proliferation in MCF-7 and MDA-MB-231 Cells after 24 h and 14 Days of Chlorpyrifos Treatment

**DOI:** 10.1021/acs.chemrestox.1c00258

**Published:** 2021-08-23

**Authors:** Paula Moyano, José Manuel Garcia, Jimena García, Adela Pelayo, Pilar Muñoz-Calero, María Teresa Frejo, Andrea Flores, Javier del Pino

**Affiliations:** † Department of Pharmacology and Toxicology, Veterinary School, Complutense University of Madrid, 28040 Madrid, Spain; ‡ Department of Legal Medicine, Psychiatry and Pathology, Medicine School, Complutense University of Madrid, 28040 Madrid, Spain; § Department of Pharmacology, Health Sciences School, Alfonso X University, 28691 Madrid, Spain

## Abstract

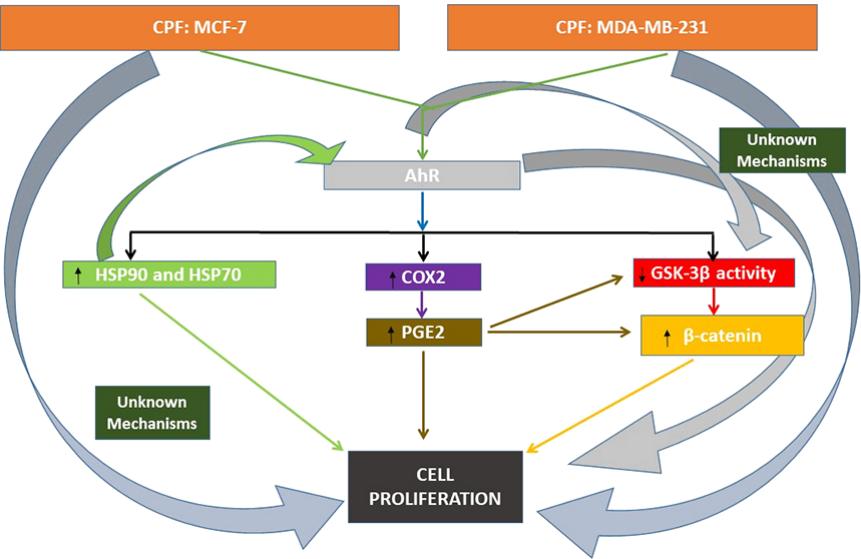

The biocide chlorpyrifos (CPF) was described to increase breast cancer risk in humans, to produce breast cancer in animals, and to induce cell proliferation in MCF-7 and MDA-MB-231 cells after 1 and 14 days of treatment. The entire mechanisms related to these CPF actions remain unknown. CPF induced cell proliferation in MCF-7 and MDA-MB-231 cells after 1 and 14 days of treatment by AhR activation through the PGE2/Wnt/β-catenin pathway and HSP90 and HSP70 overexpression. Our results reveal new information on CPF toxic mechanisms induced in human breast cancer cell lines, which could assist in elucidating its involvement in breast cancer.

Chlorpyrifos (CPF), an extensively employed biocide, was reported to generate breast cancer after repeated exposure at low doses in rats^1^ and to increase the risk of breast cancer development in women.^2^ In addition, it was reported to produce cell proliferation in human breast cancer cell lines expressing (MCF-7) or not (MDA-MB-231) estrogen receptor after unique and long-term treatment.^3,4^ However, to date the complete mechanisms through which CPF could induce this effect remain to be discovered.

CPF was reported to induce cell proliferation in MCF-7 and MDA-MB-231 cells, in part, through Wnt/β-catenin signaling disruption, aromatic hydrocarbon receptor (AhR) activation, arylacetamide deacetylase-like 1 (AADACL1, also known as KIAA1363) and acetylcholinesterase R (AChE-R) variant overexpression, reactive oxygen species (ROS) generation, and increase of ACh levels after 24 h and 14 days of treatment and only through estrogen receptor alpha (ERα) activation in MCF-7 cells after 24 h of treatment, but additional mechanisms seem to be implicated.^3,4^ Heat shock proteins (HSPs) were reported to protect against ROS, toxic misfolded or aberrant proteins, and cell death.^5^ HSP overexpression was associated with the induction of cell proliferation, migration, and invasion in different cancer types, like breast cancer.^6,7^ HSP90 and HSP70 overexpression was reported to induce cell proliferation in human breast cancer tissues^8,9^ and in MCF-7 and MDA-MB-231 cells.^10−12^ CPF was reported to induce HSP90 and HSP70 overexpression in different species after single and repeated exposure.^13−15^ Thus, CPF could also contribute to cell proliferation through the overexpression of these HSPs. In addition, HSP90 was shown to be essential in the regulation of the AhR activity, and its inhibition, or downregulation, inhibits AhR activity.^16^ However, AhR activation was shown to regulate the HSP90 and HSP70 expression among other HSPs in different species,^17,18^ so the AhR activation induced by CPF could mediate the HSP overexpression intensifying the AhR action on cell proliferation.

Prostaglandin E2 (PGE2), which is synthesized first by the rate-limiting enzyme cyclo-oxygenase-2 (COX2) and finally by the Prostaglandin E synthase, was also related to the induction of cell proliferation, migration, and invasiveness in MCF-7 and MDA-MB-231 breast cancer cells.^19^ In addition, PGE2 was reported to induce this effect through Wnt/β-catenin signaling activation by GSK-3β deactivation and β-catenin induction.^19^ CPF was reported to increase PGE2 in mouse brain samples after prenatal exposure and in rat hippocampus samples after a single treatment.^20,21^ Therefore, CPF could also induce cell proliferation through Wnt/β-catenin signaling pathway dysfunction mediated by PGE2. Otherwise, AhR was reported to regulate the synthesis of PGE2 and Wnt/β-catenin signaling pathway.^22,23^ Accordingly, we hypothesized that CPF could activate AhR, producing the upregulation of HSP90 and HSP70 proteins and the induction of Wnt/β-catenin signaling pathway, mediated by the increment of PGE2 levels, leading to cell proliferation in MCF-7 and MDA-MB-231 cells. To evaluate our hypothesis, wild type or HSP90 and HSP70 silenced MCF-7 and MDA-MB-231 cells were exposed to several CPF concentrations either alone or in combination with CH-223191 (AhR antagonist, 20 nM) and/or MF-63 (prostaglandin E synthase inhibitor, 1 μM) for 24 h or repeatedly for 14 days.

CPF toxic effects in tissues were reported to be developed by the combination of CPF and its main locally formed metabolite, chlorpyrifos oxon (CPFO).^3,4^ MCF-7 and MDA-MB-231 cells express different cytochrome P450 isoforms that metabolize CPF to CPFO.^3,4^ We used CPF for this study because we previously did not observe different actions between CPF and CPFO on cell proliferation,^3,4^ because CPF is the compound to which human population and animals are naturally exposed and because the toxic effect of CPF is produced, by CPF and CPFO, after its local metabolism in the tissues.

MCF-7 and MDA-MB-231 cell lines, used as a model of estrogen-dependent and estrogen-independent breast cancer cells, were cultured according to Moyano et al.^3,4^ Cells (passages 7–15) were seeded with complete medium (Dulbecco’s modified Eagle’s medium/F12 at 1:1 with 10% fetal bovine serum (FBS), penicillin/streptomycin, 2 mM l-glutamine, and 6 ng/mL insulin for MCF-7 or without insulin for MDA-MB-231) and left to attach for 24 h. Following attachment, phenol red-free medium with 2.5% charcoal-treated FBS was used for 24 h as experimental medium; afterward, the experimental medium was renewed, and the experimental compounds were added for either 1 day or daily with new medium for 14 consecutive days. The described conditions and times were followed for all different cotreatments.

We choose 0.01 μM to 100 μM CPF concentrations because according to the literature and previous studies they are relevant to study cell proliferation in breast cancer.^3,4^ In addition, we chose 1 μM CPF concentration, because it was the maximum concentration to induce cell proliferation.^3^ Finally, we chose CH-223191 (CH22) and MF-63 concentrations because they were the minimum concentrations that completely blocked AhR activation and PGE2 synthesis, respectively.

BrdU ELISA Cell Proliferation Assay Kit (colorimetric) (ab126556, Abcam, Cambridge, U.K.) was used, following the manufacturer’s guidelines, to elucidate the mechanisms involved on MCF-7 and MDA-MB-231 cell proliferation induced by CPF after single and repeated treatment, which were confirmed by MTT and crystal violet staining test according to Moyano et al.^3^ The GSK-3β enzymatic activity was determined by a GSK-3β Activity Assay Kit (CS0990; Sigma, Madrid, Spain) following the manufacturer’s guidelines. GSK-3β enzymatic activity values are expressed as percentages of the untreated control. PGE2 concentration in culture media was analyzed employing a commercial ELISA kit (ab133021; Abcam, Cambridge, U.K.) following the manufacturer’s protocols.

Gene expression analysis was developed employing validated primers (SA Biosciences) for mRNAs encoding β-catenin (PPH00643F), beta-actin (ACTB; PPH00073G), HSP90 (PPH00643F), HSP70 (PPH01188C), cytochrome P450 isoenzyme 1A1 (CYP1A1; PPH01271F), and COX2 (PPH01271F) according to Moyano et al.^3^ QPCR data were analyzed following the Ct (cycle threshold) method.^24^ COX2, β-catenin, HSP90, and HSP70 protein expression were determined using commercial ELISA kits (MBS264304, MBS724736, MBS2702622, and MBS012990, respectively; MyBioSource, CA, United States), following the manufacturer’s protocol. Finally, cells were transfected using siRNA (Qiagen; Barcelona, Spain) homologous to mouse HSP90 (GS3320), HSP70 (GS3303), and β-catenin (GS1499) target genes, and the transfection efficiency was measured, performing gene’s expression analysis of silenced genes, showing statistically significant reduction on the expression of these targets (Supplementary Figure 1). Our results are representative of (at least) three experiments performed for each research study in triplicate (*n* = 9). Results are presented as means ± standard error of the mean (SEM). One-way (concentration–response analysis) and two-way (gene manipulation vs treatment) ANOVA analyses followed by Tukey posthoc test were performed to identify statistically significant differences between treatments (*p* ≤ 0.05), using GraphPad Software Inc.’s (San Diego, CA, United States).

Our results show that HSP90 and HSP70 protein expression were upregulated in a concentration-dependent way, respectively, after 1 and 14 days of CPF treatment (starting at 0.1 μM) in MCF-7 and MDA-MB-231 cells (Figure 1A–D), which was correlated with the gene expression results (data not shown). CPF was reported to induce HSP90 and HSP70 overexpression in different species after single and repeated exposure,^13−15^ supporting our results. In addition, CH22 treatment reversed, in part, the overexpression of HSP90 and HSP70, noticing the mediation of AhR in the CPF upregulation of these targets (Figure 1A–D). AhR was reported to regulate the expression of different HSPs like HSP90 and HSP70,^17,18^ corroborating our data. However, other mechanisms seem to be involved. CPF was reported to activate estrogen receptor and induce NRF2 pathway,^3,4^ which were also reported to regulate HSP expression,^25^ so these mechanisms could also participate in this effect. In addition, CPF treatment of HSP90 silenced cells reversed completely the CYP1A1 overexpression induced by AhR activation (data not shown). HSP90 was shown to be essential for the AhR activity,^16^ and its inhibition blocks its activity, supporting our results.

**Figure 1:**
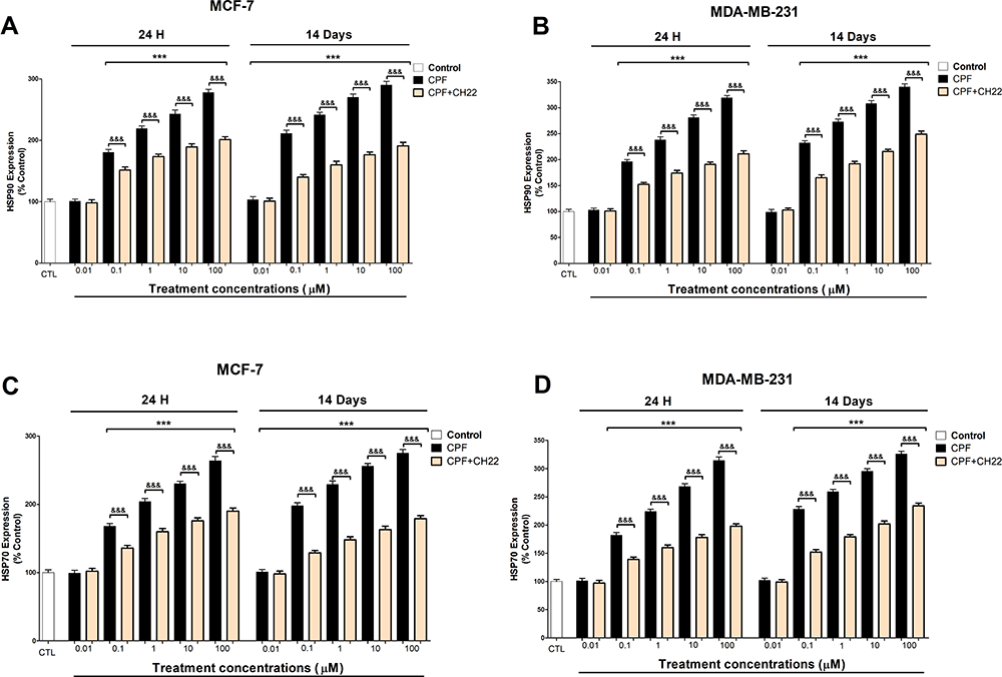
HSP90 (A and B) and HSP70 (C and D) expression analysis results. Data represent the mean ± SEM of three separate experiments from cells of different cultures, each performed in triplicate. ***p ≤ 0.001, significantly different from controls; ^&&&^*p* ≤ 0.001, compared to CPF treatment.

In addition, COX2 protein expression (Figure 2A,B), which was correlated with the gene expression results (data not shown), and PGE2 levels (Figure 2C,D) were upregulated in a concentration-dependent way after 1 and 14 days of CPF treatment (starting at 0.1 μM), and these effects were completely reversed after CH22 cotreatment with CPF. These data show that AhR mediates this effect. AhR was reported to regulate COX2 expression and PGE2 levels.^22^ CPF was reported to induce COX2 expression in MCF-7 cells through AhR^26^ and to increase PGE2 levels after single treatment,^21^ which supports our data.

**Figure 2:**
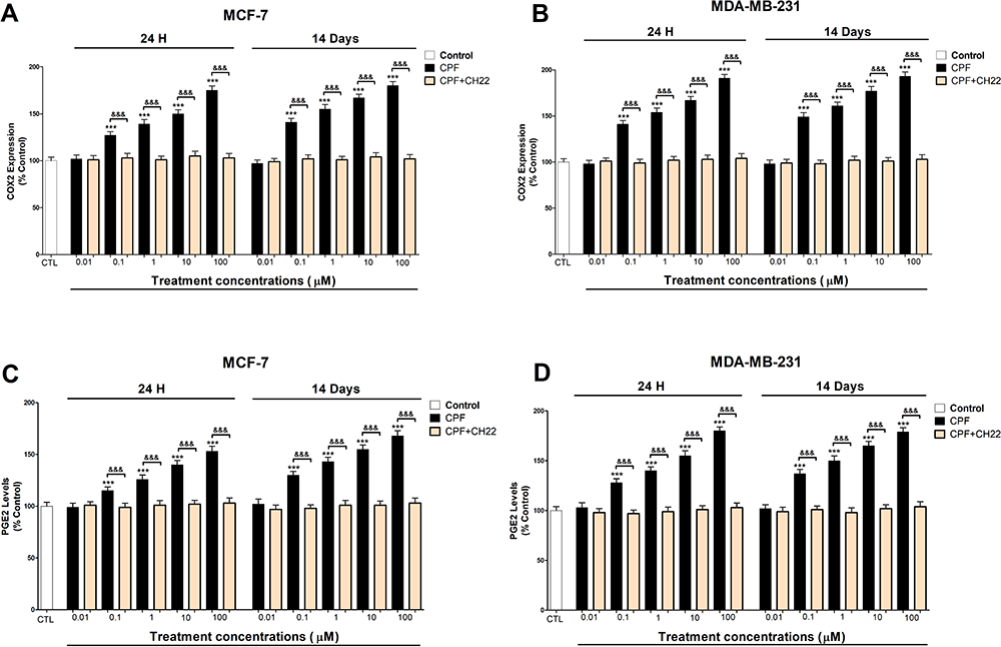
COX2 (A and B) expression and PGE2 (C and D) concentration analysis results. Data represent the mean ± SEM of three separate experiments from cells of different cultures, each performed in triplicate. ***p ≤ 0.001, significantly different from controls; ^&&&^*p* ≤ 0.001 compared to CPF treatment.

CPF also decreased GSK-3β activity and increased β-catenin expression after single and repeated treatment from 0.1 μM to 10 μM concentration, and after CH22 or MF-63 cotreatment with CPF, they were partially reversed (Figure 3A–D). However, these effects were the opposite after 100 μM (1 day) and 10 μM (14 days) concentrations, and only the CH22 cotreatment with CPF was able to partially revert these effects (Figure 3A–D). As we indicated previously,^4^ β-catenin accumulation induces cell proliferation, but its downregulation produces cell viability reduction, which may explain these opposite effects on cell proliferation/viability reduction observed after CPF treatment.^3^ In addition, our data indicate that CPF induces Wnt/β-catenin signaling pathway, in part, through the action of AhR and PGE2, which is in turn induced by AhR.^22^ PGE2 and AhR were reported to induce Wnt/β-catenin signaling activation by GSK-3β deactivation and β-catenin induction,^19,23^ supporting our results. AhR was also reported to downregulate Wnt/β-catenin pathway activity,^23^ which could explain the opposite effect on this pathway observed from 100 μM (1 day) and 10 μM (14 days) concentrations. AhR signaling pathway presents a very complex regulation, and AhR has different variants that could mediate different effects.^3^ Thus, the differences observed could result from the action of different pathway modulators or the expression of different AhR variants, depending on the concentration. Otherwise, additional mechanisms seem to be involved in the observed effects. We previously described that CPF also mediated this effect through ROS generation,^4^ and we hypothesized that other targets, which were reported to be affected by CPF-like protein kinase C and histone deacetylase 1 that regulate GSK-3β,^27,28^ could also contribute to this effect.^4^

**Figure 3:**
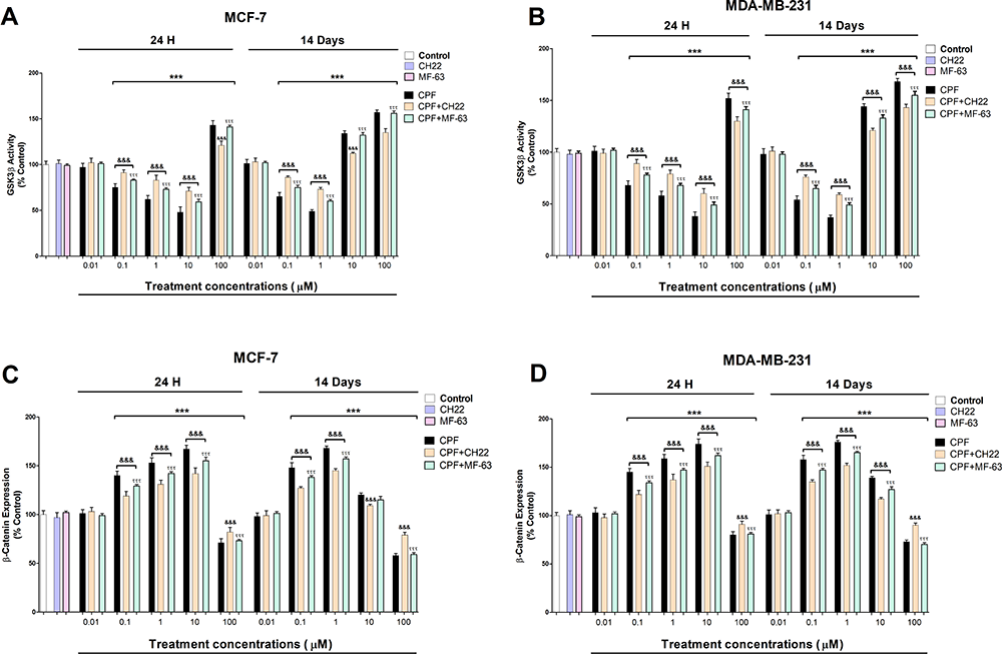
GSK3β (A and B) activity and β-catenin (C and D) expression analysis results. Data represent the mean ± SEM of three separate experiments from cells of different cultures, each performed in triplicate. ***p ≤ 0.001, significantly different from controls; ^&&&^*p* ≤ 0.001 compared to CPF treatment.

Lastly, BrdU results show that CPF produced proliferation of MCF-7 and MDA-MB-231 cells after 1 and 14 days of treatment (Figure 4A,B), which was previously described.^3,4^ These results were partially reverted after CPF treatment of simultaneously HSP90 and HSP70 silenced cells, of β-catenin silenced cells, or after cotreatment with CH22 or MF-63, which shows that HSP90, HSP70, AhR, PGE2, and β-catenin mediated the cell proliferation observed after CPF treatment alone. These data were corroborated by an MTT test (data not shown). We previously showed that CPF mediated this effect through AhR and Wnt/β-catenin signaling pathway activation.^4^ In addition, PGE2 was reported to induce cell proliferation in MCF-7 and MDA-MB-231 breast cancer cells.^19^ HSP90 and HSP70 overexpression was reported to induce cell proliferation in MCF-7 and MDA-MB-231 cells.^10−12^ Therefore, these data support our results. In addition, the CH22 and MF-63 concomitant treatment of simultaneously HSP90, HSP70, and β-catenin silenced cells induced a greater reversion, but it was still incomplete, suggesting that other mechanisms may be implicated. In this regard, we previously described that CPF mediates this effect through the AChE-R variant and KIAA1363 overexpression, increase of acetylcholine levels, activation of estrogen receptor, and ROS generation;^3,4^ therefore, all these mechanisms together could be contributing to this effect, but we cannot discard other mechanisms. Paraoxonase overexpression was reported to induce tumor growth.^29^ CPF exposure was shown to upregulate paraoxonases expression;^30^ therefore, this mechanism could also contribute to the effect observed.

**Figure 4:**
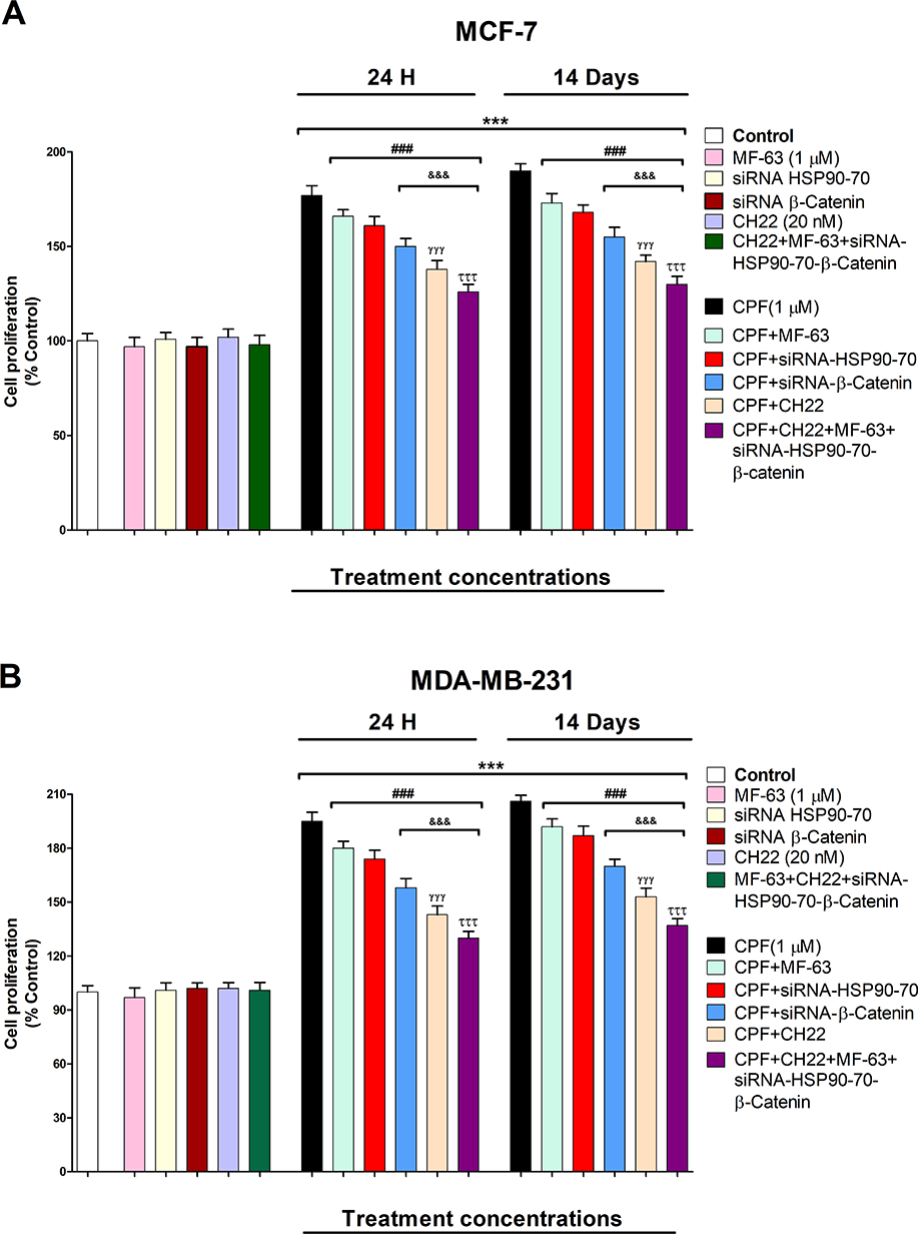
CPF (1 μM) effect on the proliferation of wild-type or single or simultaneous HSP90-70 and β-catenin-silenced MCF-7 (A) and MBA-MB-231 cells (B) cotreated with or without CH22 (20 nM) and with or without MF-63 (1 μM). Data represent the mean ± SEM of three separate experiments from cells of different cultures, each performed in triplicate. ***p ≤ 0.001 compared to the control; ^###^*p* ≤ 0.001 compared to CPF treatment; ^&&&^*p* ≤ 0.001 compared to CPF treatment of HSP90–70 silenced cells; ^γγγ^*p* ≤ 0.001 compared to CPF treatment of β-catenin silenced cells; ^τττ^*p* ≤ 0.001 compared to CPF cotreatment with CH22.

These data show that CPF induces cell proliferation in MCF-7 and MDA-MB-231 cells after 1 and 14 days of treatment by AhR activation through PGE2/Wnt/β-catenin pathway and HSP90 and HSP70 overexpression. These data may help explain the CPF action in the proliferation of breast cancer cells. Further studies should be performed to determine if all mechanisms that we described mediate cell proliferation or whether additional mechanisms are involved and to confirm that these mechanisms mediate cell proliferation *in vivo*. Our results are of interest because they supply novel insights on the mechanisms that mediate cell proliferation induced following CPF exposure in human breast cancer cell lines.

## Supporting Information Available

Supplementary Figure 1: Transfection efficiency for single or simultaneous siRNA targeting HSP90, HSP70, and β-catenin (PDF)
