# Supplementary material for: Aryl Hydrocarbon Receptor Activation Produces Heat Shock Protein 90 and 70 Overexpression, Prostaglandin E2/Wnt/β-Catenin Signaling Disruption, and Cell Proliferation in MCF-7 and MDA-MB-231 Cells after 24 h and 14 Days of Chlorpyrifos Treatment
Source: Chem Res Toxicol. 2021 Aug 23;34(9):2019–23. doi: 10.1021/acs.chemrestox.1c00258 (PMC9132385; doi:10.1021/acs.chemrestox.1c00258)
Supplement: Supplementary file 1 — tx1c00258_si_001.pdf [file tx1c00258_si_001.pdf]

# Supporting Information

# Aryl hydrocarbon receptor activation produces heat shock protein 90 and 70 overexpression, prostaglandin E2/Wnt/β-catenin signaling disruption and cell proliferation in MCF-7 and MDA-MB-231 cells after 24 h and 14 days chlorpyrifos treatment.

Paula Moyano[*1], José Manuel Garcia[1], Jimena García[3], Adela Pelayo[2], Pilar Muñoz-Calero[2], María Teresa Frejo[1], Andrea Flores[1], and Javier del Pino[*1]

[1]Department of Pharmacology and Toxicology, Veterinary School, Complutense University of Madrid, 28040-Madrid, Spain.
[2] Department of Legal Medicine, Psychiatry and Pathology, Medicine School, Complutense University of Madrid, 28040, Madrid, Spain
[3] Department of Pharmacology, Health Sciences School, Alfonso X University, 28691, Madrid, Spain.

**Supplementary Figure 1.**

**A**

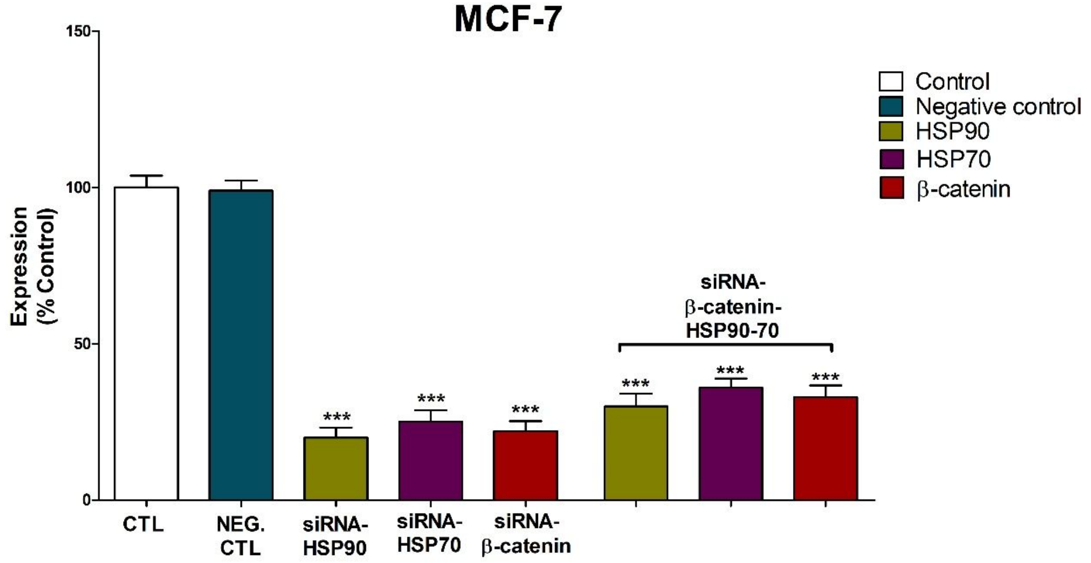


**B**

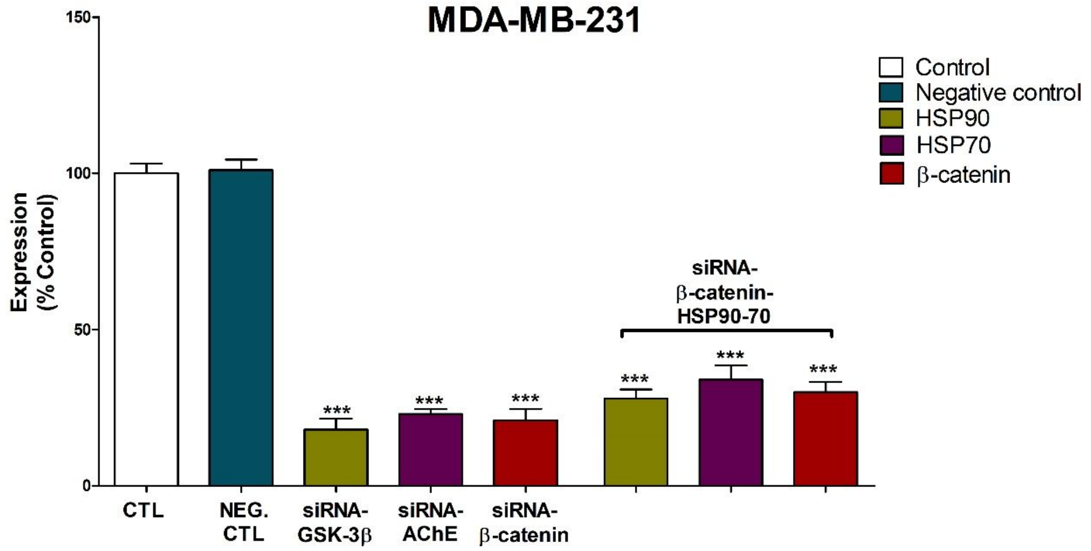


**Supplementary Figure 1.** Transfection efficiency for single or simultaneous siRNA targeting HSP90, HSP70, and β-catenin. $^{***}p \leq 0.001$ compared to the control.
